# Amputation and rotationplasty in children with limb deficiencies: current concepts

**DOI:** 10.1007/s11832-016-0788-7

**Published:** 2016-11-08

**Authors:** Ralph Sakkers, Iris van Wijk

**Affiliations:** 1 Department of Orthopaedic Surgery, Wilhelmina Children’s Hospital, University Medical Center Utrecht, P.O. Box 85090, 3508 AB Utrecht, The Netherlands; 2 Brain Centre Rudolf Magnus and Centre of Excellence for Rehabilitation Medicine, De Hoogstraat Rehabilitation, University Medical Center Utrecht, Utrecht, The Netherlands

**Keywords:** Limb deformity, Limb deficiency, Postaxial hypoplasia, Syme amputation, Rotationplasty

## Abstract

**Purpose:**

Amputations and fitting surgery have a long history in children with limb deficiencies. With the current developments in limb reconstruction and new techniques in prosthetics, the indications for amputation and fitting surgery might have shifted, but still have a very important role in creating high functional performance, optimal participation and quality of life. The purpose of this current concepts article is to give an overview of the indications, dilemmas and technical considerations in the decision-making for amputation and fitting surgery. A special part of this overview is dedicated to the indications, variations and outcomes in rotationplasties.

**Methods:**

The article is based on the experience of a multidisciplinary reconstruction team for children with complex limb deficiencies, as well as research of the literature on the various aspects that cover this multidisciplinary topic.

**Results:**

For those children with a more severe limb deficiency, reconstruction is not always feasible for every patient. In those cases, amputation with prosthetic fitting can lead to a good result. Outcomes in quality of life and function do not significantly differ from the children that had reconstruction. For children with a postaxial deficiency with a femur that is too short for lengthening, and with a stable ankle and foot with good function, rotationplasty offers the best functional outcome. However, the decision-making between the different options will depend on different individual factors.

**Conclusions:**

Amputations and rotationplasties combined with optimal prosthesis fitting in children with more severe limb deficiencies may lead to excellent short- and long-term results. An experienced multidisciplinary team for children with complex limb deficiencies should guide the patient and parents in the decision-making between the different options without or with prosthesis.

## Introduction

Limb deformities in childhood can be congenital, as in children with pre- or postaxial hypoplasia [tibial deficiency, fibular deficiency, proximal focal femoral deficiency (PFFD) and ray defects of the foot], or acquired, as in children with progressive deformities due to sepsis or trauma involving growth plates or tumours and sequelae of the resection of tumours. In some of these children with limb deformities, the dilemma of reconstruction versus amputation will arise. Indications for reconstruction versus amputation will not only depend on the technical possibilities and the prognosis on functional outcome, but also on the wishes of the patient and the parents, the amount of time to invest in youth, the perceived quality of life during youth and in adulthood, and the cultural differences in acceptance of cosmetic appearance. A special type of functional reconstruction through a rotationplasty is another possibility in a specific group of children, but this highly functional reconstruction will not always be acceptable from a cosmetic point of view in the patient groups with different cultural backgrounds.

This article will focus on the different aspects that play a role in the decision-making for amputations as well as the anatomical requirements (weight-bearing, stump length) for adequate prosthetic fitting at the different ages in children with congenital limb deficiencies. Indications for rotationplasties will also be discussed with and without an anomaly of the hip joint.

## Primary goals in reconstruction surgery of the lower limb

The primary goal in reconstruction of a lower limb is to achieve an optimal functional outcome for each individual patient. At the end of growth, the pelvis should be horizontal in stance, the axis deformations minimalised, with the patient standing on his/her own feet or with the prosthesis on. Comfortable sitting, preferably with the knees with or without prosthesis at the same distance from the hip, compatible with most chairs should be aimed for. A good endurance in walking activities, by creating a stable weight-bearing lower extremity, and active participation in sports are other important goals. Last but not least, cosmetic appearance should be taken into account.

## The dilemmas in reconstruction versus amputation

The configuration, stability and range of motion of the ankle and foot joints are the main determinants for adequate weight-bearing on the foot. The amount of rays in a foot is of lesser importance. For example, a plantigrade one-ray foot with a stable ankle joint and a minor leg length discrepancy can have a good weight-bearing capacity and a better functional outcome than a five-ray unstable valgus foot with a synostosis of talus and calcaneus in a parallel position and a complete fibular deficiency with a leg length discrepancy of more than 50%. On the other hand, equal leg length with a partial transverse deficiency of the forefoot and a stable ankle joint can result in adequate weight-bearing without shoes, but can raise such fitting problems for shoes that fitting surgery by amputation is asked for by the patient for optimal prosthesis fitting in combination with regular shoes. The advanced dynamic properties of prosthetic feet in contrast to the aplastic own foot could also contribute to the wish for an amputation.

The leg length discrepancy is another important item in decision-making. In general, leg length discrepancies in one bone larger than 30% are considered too much for reconstruction by lengthening of bone and soft tissues, especially when combined with significant joint abnormalities [1]. With modern techniques, like the newer reconstructions for the ankle, knee and hip joint, and the possibility for a more patient friendly intramedullary bone lengthening from a certain age [2, 3], the indications for lengthening are expanded. If stable joints are successfully created, larger leg length discrepancies sometimes require up to four lengthening procedures during youth starting from around the age of 2 years and might be repeated every 3–6 years [1, 4]. With a healing index ranging between 1.2 and 2 months/cm [2, 4, 5] and a lengthening of between 3 and 8 cm per lengthening of a bone per period of lengthening [1, 3, 4], the lengthening procedures might require a significant amount of time and effort by both the patient and the parents, and may interfere with the social life of the patient in his/her important childhood and adolescent years. Dealing with these interventions including dealing with pain might be too much of a burden for certain families. In our opinion, proper screening and education of both the child and the parents is necessary. The advantages and disadvantages of the different choices (amputation, reconstruction and lengthening, or prosthetic fitting without surgery) should be elicited in terms of the amount of time and effort to invest, the physical and psychological impact of the proposed procedure and the expected impact on quality of life during childhood and the quality of life in the long run. The role of other families who went through the same procedures is of utmost importance [6]. Costs of repeated surgical procedures are expensive, but in the long run, it is probably less expensive than lifelong prosthetic provisions [7]. However, functional outcome in youth and adulthood as well as a personal satisfactory cosmetic outcome in youth and adulthood are the major important determinants. In the scarce literature on long-term outcomes of patients with significant limb deformities that were lengthened with reconstruction and those patients that had a primary amputation, there was no significant difference in outcome in both performance as well as quality of life [8–12]. Limitations of these studies are heterogeneity of the groups. On the other hand, studies and institutions that report improved outcomes of newer surgical techniques lack a current objective comparison with amputation groups. Although surgical techniques have improved, prosthetic techniques have also improved in such a way that even in top-level sports, the difference is small [13, 14]. The current Olympic record (2012) for 100 m running is 9.63 s held by Usain Bolt versus 10.9 s for the “Blade Runner” Jonnie Peacock at the Paralympics (which equals 8th place in the 2012 Olympic final). The current Olympic record (2012) for the high jump is 2.38 m by Ivan Ukhov versus the record (2012) of 2.12 m by Maciej Lepiato. The current world record for the long jump is 8.95 m by Mike Powell (1991) versus 8.40 m for the “Blade Jumper” Markus Rehm (2015).

With all the different dilemmas in decision-making, the final choice remains an individual one, especially in those patients in the grey zone of indication for reconstruction or amputation [15]. This choice will be dependent on the individual’s important parameters for his/her quality of life. These parameters are influenced by previous experiences, personal beliefs and adjustment capacities, and will correlate strongly to the psychological well-being, self-esteem and social circumstances of the patient and their parents [16, 17]. Therefore, a multidisciplinary treatment team, with sufficient knowledge of all important factors (physical, psychological, social) that play a role, should preferably guide the decision-making in order to get a final individualised and optimal short- and long-term outcome [18]. For a complete education, adequate photographic and video material is necessary, and contacts with individual patients and parents to share experiences, and contacts with the patient organisation should be offered. These contacts are usually much appreciated and useful for the parents to get a better idea of the functional outcomes and cosmetic appearance by seeing real children with different solutions.

In case of a choice for a primary amputation of the foot and alignment of the tibia, the preferred timing is before walking age in most cases, i.e. around the age of 1 year [19]. Sometimes, especially in cases in which the leg length discrepancy is predominantly in the femoral part and the quality of the ankle joint is good, the decision for amputation or rotationplasty will be postponed to a later age, depending on the other choices made for reconstruction.

## Technical considerations in amputations and fitting surgery

One of the issues to address in amputation and fitting surgery is the choice between weight-bearing and non-weight-bearing solutions. If possible, a weight-bearing level of amputation is the first choice. Advantages of a weight-bearing stump are a less aggressive fitting of the prosthesis, distal loading through the retained heel pad, which seems to give more stability and less complications such as bony overgrowth of the bony end with possible ulceration of the skin [7, 19, 20]. In childhood, there is a possibility to stand on the stump without prosthesis, which can be an advantage in, for example, the shower or the swimming pool. Despite the progressive leg length discrepancy during growth, the weight-bearing capacities are an advantage with transfers or walking short distances in the house.

The most often used weight-bearing level of amputation below the knee is the Syme and the Boyd amputations [7, 20]. Both procedures are, in fact, ankle exarticulations that do not disturb the epiphysis. Syme described his technique of exarticulation of the foot in 1842, in which he covered the end of the tibia and fibula with the strong skin of the heel pad. The main problems described with Syme’s technique are possible posterior migration of the heel pad, heel pad slough and fitting problems. However, in general, these issues are reported as not significant [9]. The Boyd amputation, described in 1939, is also a disarticulation through the ankle, but in this procedure, the posterior part of the calcaneus is preserved and fused to the distal tibia [21, 22]. The heel pad remains attached to the posterodistal part of the calcaneus, preventing heel pad migration after solid fusion of the posterior part of the calcaneus to the distal tibia. From a personal experience, a Syme amputation gives a more natural placement of the heel pad in those children that have a severe equinus hind foot with a low height of the talus calcaneus (synostosis) bone in fibular deficiency, where the heel pad just fits over the end of the tibia with or without fibula. On the other hand, an exarticulation of a foot with a normal height of the hind foot at a later age can benefit from the extra length of the part of the calcaneus distal from the tibia for a natural placement of the heel pad.

When a transtibial amputation cannot be avoided, osseous overgrowth is a well-known problem. Different techniques have been developed to prevent an open marrow space at the end of the bone, with various degrees of success [23, 24]. Especially techniques that provide an osteocartilaginous ending to the end of the bone, such as, for example, transplanting a fibular head or a metatarsal head in the end of the tibia seem to decrease the percentage of overgrowth from 50% to around 0–10% [24]. Planning on optimal stump length at adult age including the calculation of remaining growth in the growth plates at the timing of reconstructions and fitting surgery is important. The minimum stump length for adequate prosthesis fitting in below-knee amputations is 10–15 cm to preserve enough leverage for power and control of the prosthesis [25, 26]. On the other hand, the leg length difference after amputation might be too small to be able to build in a more advanced prosthetic foot in adolescence and adulthood. The optimal leg length difference is currently between 12 cm (e.g. Vari-Flex Junior) and 18–30 cm (sports foot like Cheetah Xplore) for these more dynamic prosthetic feet that are believed to have several advantages for the consumer [13, 25].

In transtibial amputees for example, there seems to be a slight trend towards a greater stride length when walking with the Flex-Foot in comparison to the SACH foot [25, 27, 28]. When walking speed was increased or when subjects were walking on a decline or incline treadmill, the energy cost was lower with an energy-storing foot than with the SACH foot [25, 29]. Most prosthetic companies have a special Syme-type prosthetic foot for adults that need a 5–6 cm average build height. Newer materials such as glass fibre composites in the RUSH® foot promise to have more dynamic properties and durability even in the low build-height feet for active kids and adults.

**Fig. 1:**
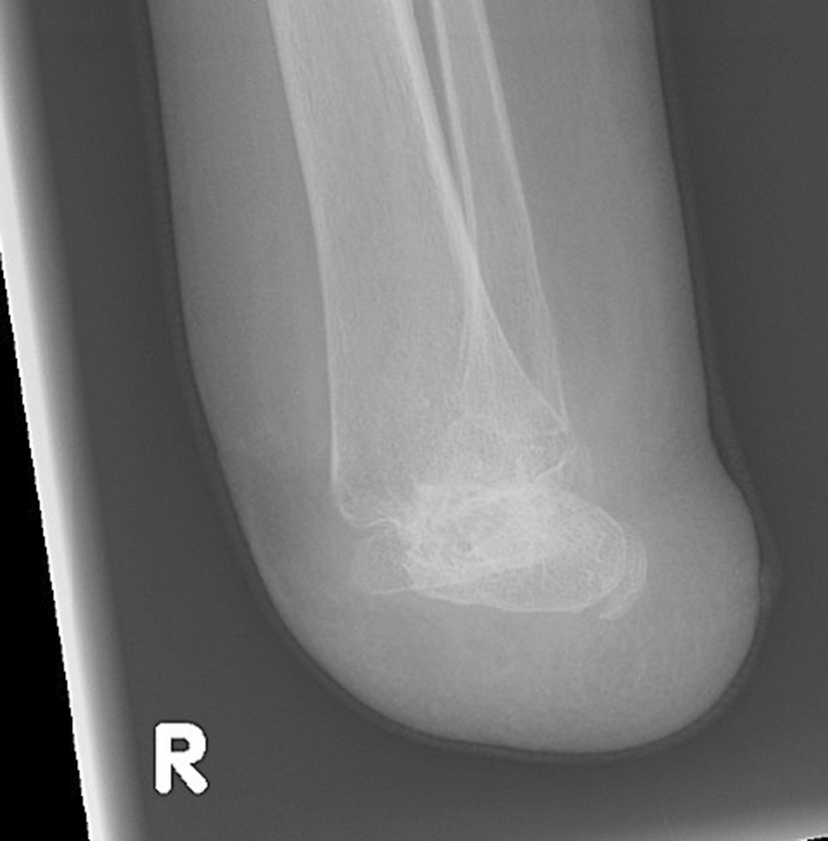
Typical example of a Boyd amputation for optimal fitting in a below-knee prosthesis

**Fig. 2:**
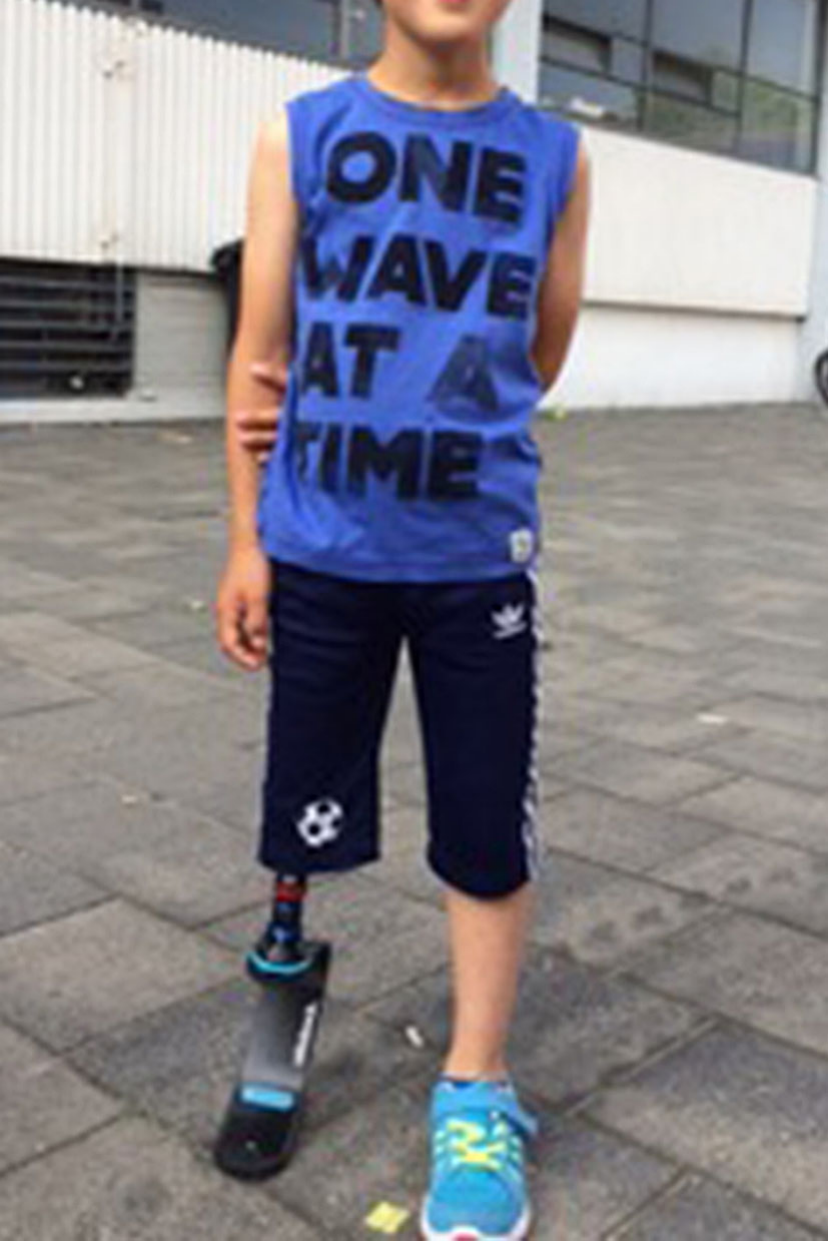
Five-year old boy with a congenital knee disarticulation fitted with a blade for running

Thus, if possible, a weight-bearing level of amputation like a Syme or Boyd amputation, combined with an epiphysiodesis to create the optimal leg length difference for the more advanced prosthetic feet seems to be the best option. This option can only be created during the growth period in childhood (Figs. 1 and 2).

## Technical and functional possibilities with rotationplasties

Indications for rotationplasties in patients with postaxial hypoplasia are usually seen in patients where PFFD is the dominating factor and the ankle and the foot have a (near) normal anatomy and function. Mild PFFD may be treated with guided growth, alignment surgery and/or lengthening techniques. However, in femurs with length discrepancies of over 30–50%, lengthening might not be possible and one has to find other ways for a better functional outcome. An extra important goal is comfortable sitting, preferably with the knees at the same distance from the hip, and a length compatible with most chairs.

Borggreve is known to be the first surgeon to create a functional knee joint by excising the original knee with part of the distal femur and proximal lower leg with sparing of the neurovascular structures. With subsequent 180° rotation of the distal part of the lower leg with a well-functioning ankle and foot and fusing this part to the proximal part of the femur, he created a new ‘knee’ joint with the ankle for fitting into a below-knee prosthesis without the foot sticking out. This procedure was performed in a 12-year-old boy who had a stiff knee from tuberculosis [30]. In 1937, Van Nes performed rotationplasty in a patient who had PFFD and this procedure became popular in the 1940s and 1950s. The procedure became known as the Van Nes rotationplasty [6, 8, 19, 31–34].

The Van Nes rotationplasty is a creative solution for PFFD with high functional outcomes and high outcomes of quality of life, despite reported reduced symmetry in stance and reduced endpoint and maximum excursions [8]. Modifications have been made to prevent the derotation of rotated segment by de-attaching all muscle tendon units and re-attaching these 180° opposite of the joint and to address different levels of resection of bone, including a tibial rotationplasty [34–36].

Winkelmann made a modified classification of rotationplasties on the basis of which part of the leg had to be resected in oncology [37, 38]. The procedure in AI and AII lesions resulted in a femur with a normal hip joint and an ankle as the knee joint. The procedure in the BI and BII lesions have a 180° rotated knee fused to the pelvis after resection of the largest part of the femur without or with part of the pelvis. The knee is fused at the level of the hip joint and points downward to the floor, acting as a hip joint with one axis. Flexion of the ‘hip’ occurs with flexion of the knee and extension of the ‘hip’ with extension of the knee at the level of the pelvis. With lesion BIIIa, the total femur is removed, the rest of the leg is rotated 180° and the tibial plateau is placed in the acetabulum. In children below the age of 10 years, the (original) lateral part of the tibia plateau can even be remodelled to a new ball joint in the acetabulum.

Projecting this classification and procedures on patients with PFFD, the more severe PFFD patients in which lengthening procedures will not produce an adequate length of the femur can be divided into two groups: those patients with a functional hip joint and those patients without a functional hip joint.

In general, the group of patients with PFFD with a functional hip joint might need a procedure to optimise the hip apart from other reconstructive procedures. Part of these patients will need an acetabular and or femoral correction osteotomy for a better stability and long-term outcome of the hip. The next question is if and how to create a functional length from hip to knee. Some patients accept a short femur and difference in knee height and use a lengthening prosthesis for walking. Other patients prefer a knee arthrodesis and the foot at the level of the knee to fit into a prosthesis in which the foot can actively control the prosthesis with a hinge at the level of the opposite knee of the normal leg [39]. This solution has the disadvantage that the foot is sticking out at the level of the knee with problems of clothing and cosmetic issues. Therefore, some patients choose for a Syme or Boyd amputation at the level of the opposite knee joint. This gives a much better cosmetic appearance, however at the cost of a functional joint at the level of the knee and, therefore, a lower functionality of the total leg and a higher energy expenditure during walking and other activities [39]. The best functional procedure that can be offered is the rotationplasty comparable with Winkelmann AI and Winkelmann AII result in combination with a specific rotationplasty prosthesis [19, 33, 34, 36, 38].

For the group of patients without a functional hip joint, the main goal is also to create a functional upper part of the leg that can be fitted with a below-knee prosthesis. Several solutions have been created. This group can be divided into a group with a knee joint and a group without a knee joint.

In the group with a knee joint and a short distal femur, the patient can be fitted with a prosthesis with ischial support. Sometimes, it is useful to perform an early epiphysiodesis of the distal femur to keep this part as short as possible for better fitting of the prosthesis during growth. Some of these patients will choose for a knee fusion with or without a Syme or Boyd amputation.

Also in this group, different types of rotationplasties have been described. One method is femoropelvic fusion with the distal femur parallel with the ground, resulting in a knee acting as a one-axis hip joint with 90° flexion (full extension of the knee at the pelvic level) [40]. Epiphysiodesis of the short distal part of the femur was needed in some of the cases to prevent growing of the distal part of the femur in the anterior direction and some patients had a rotationplasty in the lower leg or a Syme amputation [41]. The method with resection, rotationplasty and femoropelvic fusion, in which the short distal femur is rotated 180° and fused to the pelvis with the knee pointing downwards to the floor and the knee acting as a hip joint with one axis, similar to the procedure for Winkelmann BI, can be used in PFFD without a hip joint. The method similar to the procedure for Winkelmann BIIIa in order to create a ball joint at the articulation of the original lateral part of the tibia plateau and the acetabulum can be used in young patients [42]. Prerequisites are a functional ankle and foot that can be fitted in a rotationplasty prosthesis.

**Fig. 3:**
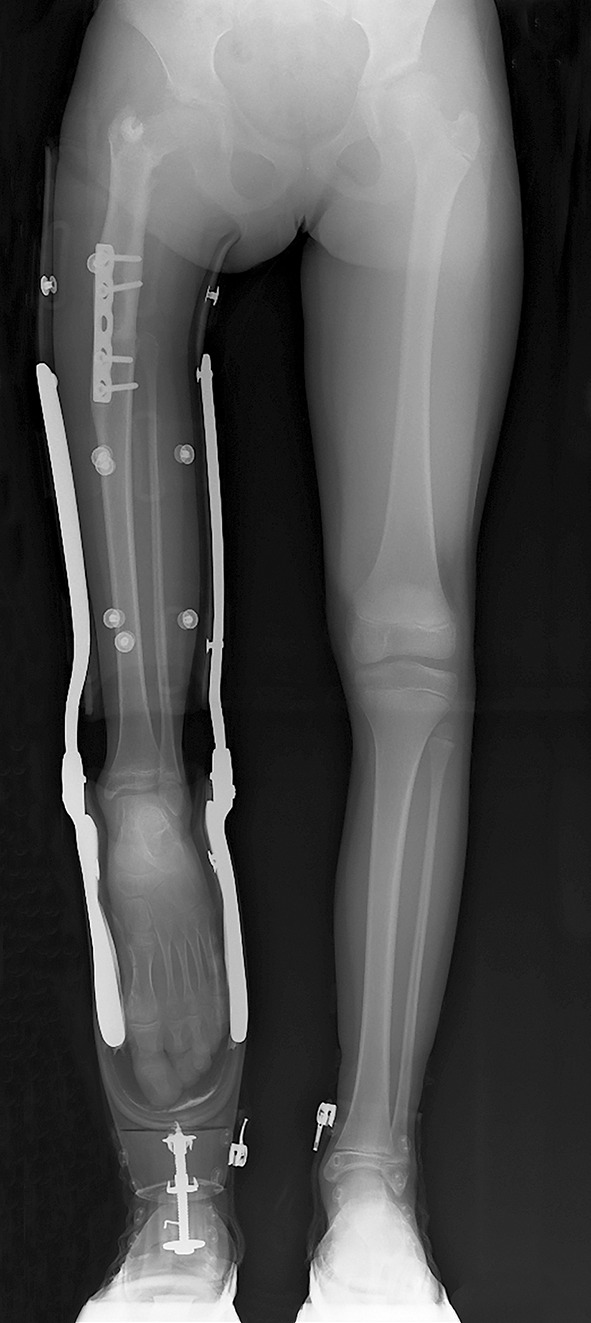
Nine-year-old girl with rotationplasty (procedure as for Winkelmann A1) for postaxial hypoplasia with dominant proximal focal femoral deficiency (PFFD) component. Length is calculated on the remaining growth of the* left* leg

**Fig. 4:**
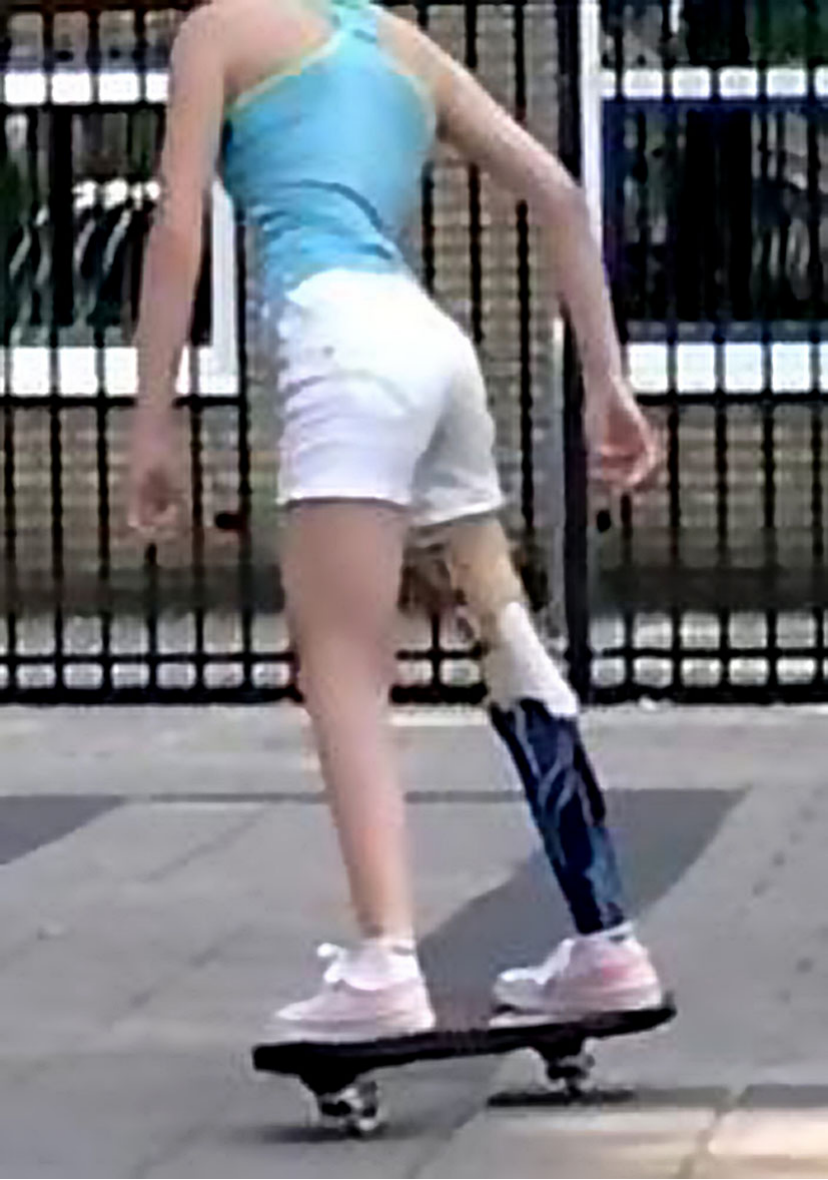
Optimal function with a rotationplasty prosthesis on a rip stick after further growth of the left femur at the age of 14 years, with the 180° rotated ankle joint at a level a few centimetres above the contralateral knee joint

**Fig. 5:**
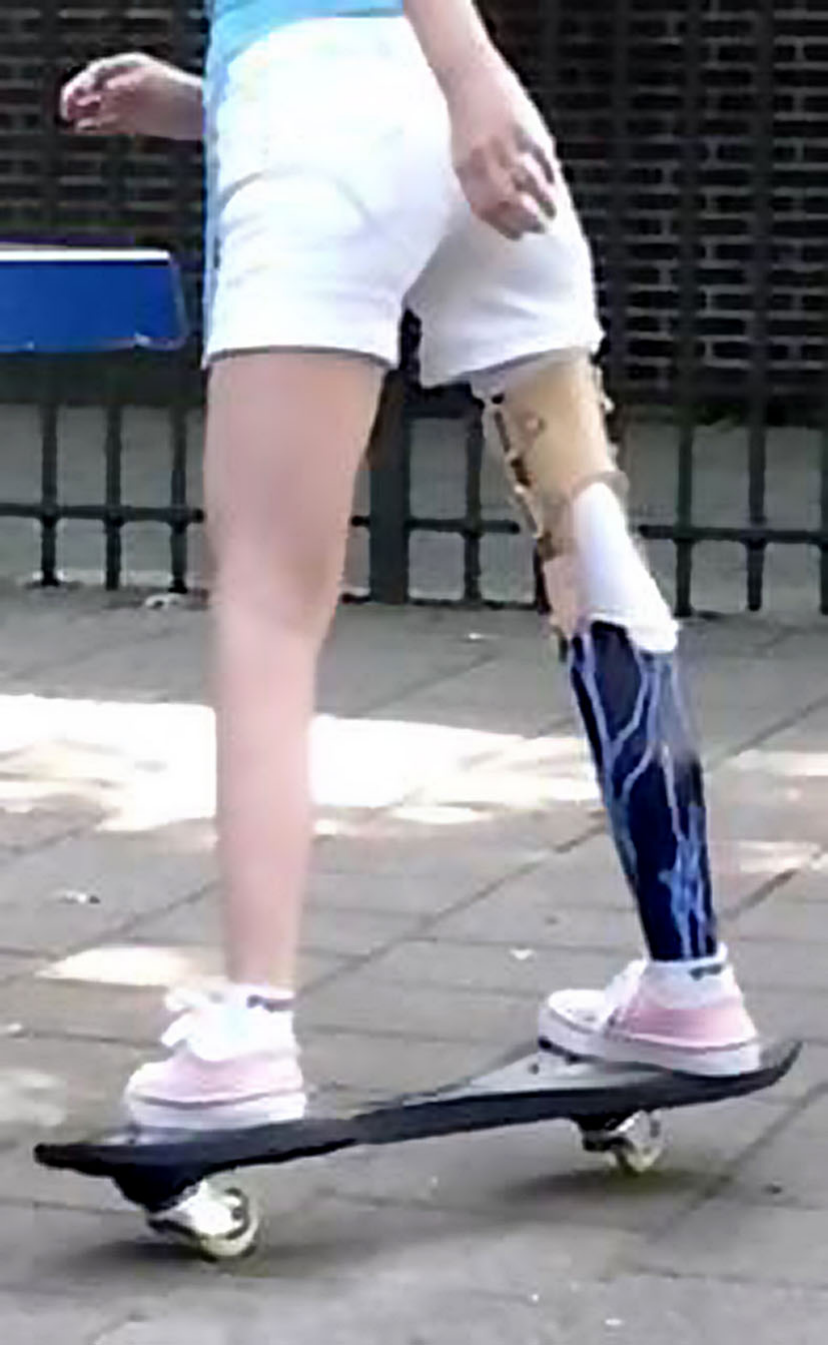
Optimal function with a rotationplasty prosthesis on a rip stick after further growth of the left femur at the age of 14 years, with the 180° rotated ankle joint at a level a few centimetres above the contralateral knee joint

In all procedures, the heel should end a few centimetres short compared to the level of the contralateral patella with sitting in order to have equal thigh length on both sides with sitting with the prosthesis on the leg. Although all reports on quality of life scales in patients with a Van Nes rotationplasty and modifications generally do not show any difference with a control population [43], the biggest controversy for this procedure for some patients is the cosmetic appearance without prosthesis, which makes this procedure not acceptable for every suitable candidate (Figs. 3, 4 and 5).

## Conclusion

Amputation and fitting surgery in children with severe limb deficiencies can lead to very functional and satisfactory results. Even in those children in whom reconstruction is possible, an individual cost–benefit ratio should be made. When the investment in time and effort for reconstruction is very high, patients and parents might also choose for amputation and fitting surgery. Functional outcome and quality of life might be equal or even better in individual cases. A rotationplasty can be the best long-term functional option in patients with a postaxial deficiency with a functional ankle and foot, and a femur that is too short for lengthening. The biggest controversy in this highly functional option is the cosmetic appearance without prosthesis. In general, multiple issues regarding surgery, prosthetic prescription and rehabilitation arise in decision-making in patients with more severe limb deficiencies. The overall purpose is to create a high functional performance, optimal participation and quality of life, and outcome is influenced by multiple (physical, psychosocial and social) factors. The incidence of limb deficiencies is low and, therefore, knowledge should be centred. The impact on childhood and adult life can be high, and the choices to be made should be guided by an experienced multidisciplinary team that is also able to evaluate new innovative techniques in both surgery and prosthetic design in a broad reference spectrum.
